# Rapid Rescoping and Adaptation: An Evaluation of the Impact of COVID-19 on Postgraduate Medical Student Research Projects

**DOI:** 10.1007/s40670-023-01777-0

**Published:** 2023-03-30

**Authors:** Joanne Hart, Rajneesh Kaur, Richmond Jeremy

**Affiliations:** School of Medicine, Faculty of Medicine and Health, University of Sydney, Sydney, Australia

**Keywords:** Research projects, Medical students, COVID-19, Research education, Scholarly projects

## Abstract

The COVID-19 pandemic has adversely affected tertiary science and medical education, with significant impact on research-related activities. Research projects are a mandatory requirement of the Doctor of Medicine (MD) Program at the University of Sydney, and medical student projects are delivered across multiple sites in metropolitan and rural New South Wales, Australia. Several cohorts of medical students had projects that were affected by COVID-19. The aims of this study were to determine the impact of COVID-19 on medical student research projects and describe the measures taken to rescope projects, to support students in meeting the learning objectives of the program. Mandatory submission statements for all medical student research project scientific reports for 2020–2022 were examined for reports of the effect of COVID-19 on the project, including COVID-19 related delays, downsizing and the need to change research project types. During the study period, a total of 760 student reports were submitted, of which 217 (28.7%) were affected by COVID-19. About 50% were significantly delayed, 30% were downsized, and 6% required completely new projects. Rescoping arrangements implemented facilitated the successful completion of projects. Overall, the final student grades for the research projects were unaffected by COVID-19 or the related project rescoping. Whilst significantly impacted by COVID-19, medical student research projects were completed with provision of appropriate rescoping plans and academic support. Ensuring projects have a documented contingency plan secured projects as the pandemic progressed and will be a useful safeguard for all future project delivery.

## Introduction

The COVID-19 pandemic and associated quarantine restrictions have markedly disrupted many activities of life, including higher education. In Sydney, Australia, from early March 2020 research and educational activities were severely impacted [1, 2] with further significant restrictions imposed from June-October 2021 [3]. Public health orders to stay at home led to closure of university and research facilities, and the Australian international border was closed to all but returning citizens. Transport services were markedly affected and supply of laboratory reagents and consumables limited [1]. With the wide uptake of vaccination, restrictions eased, however newer COVID-19 variants continue to arise, and further significant waves of infection may be anticipated [4].

### Educational Context

As part of the University of Sydney Postgraduate Doctor of Medicine (MD) Degree Program, all medical students undertake an individual, compulsory 320-h research project, supervised by academic staff members, including clinicians, biomedical and public health researchers. The scope of the research work includes devising research question(s) and/or hypotheses to test, conducting a literature review, learning the appropriate research methodology skills, collecting and/or analysing data and writing up a scientific report. The learning objectives of the MD Project support students in developing fundamental research skills by conducting a research project (Table 1).

**Table 1:** Learning objectives of the research project program

**MD Project learning objectives**	
**1**	Formulate a research question, hypothesis, or issue for investigation
**2**	Appraise the ethical issues pertaining to the project
**3**	Identify, obtain, and integrate existing knowledge relevant to the research question or hypothesis
**4**	Organise and conduct a research project
**5**	Collect and analyse data and logically present the findings
**6**	Prepare a scientific report that draws appropriate conclusions from the findings, recognises the strengths and limitations of the design and methods of the project and considers the findings considering current knowledge in the area

The scope of MD Projects offered is broad and includes clinical studies, projects in biomedical science, epidemiology, public health, medical education, bioinformatics, information technology, policy, law, and ethics. The range of possible project types is shown in Table 2.

**Table 2:** Research Project types

**Project type**	**Description**
**Prospective studies**	Clinical or health related, require participant recruitment
**Retrospective studies**	Medical record audits, collect data from existing records
**Database analysis projects**	Analysing an existing clinical or health related dataset
**Laboratory projects**	Laboratory based, biomedical or basic sciences
**Literature reviews**	Narrative, scoping or systematic reviews
**Study protocol development**	Clinical or laboratory projects, includes preparing the ethics application documents where required
**Product development projects**	Includes a needs analysis and scholarly development of patient or medical education resources

The MD Research Project program is overseen by the MD Project team, including academic and administrative staff, and runs over eight Clinical School locations (metropolitan and rural) and multiple research institutes. Projects are overseen by academic Research Co-ordinators at each location, and each student has an academic project supervisor. The supervisors manage up to six student projects. Each student submits a written report of up to 3000 words, which is independently assessed by academic staff with reference to a defined marking rubric.

### Effect of COVID-19 on Medical Student Projects

Medical students’ attendance at Clinical Schools was restricted substantially during 2020 and 2021, when students were at different phases of the research project, with both year 2 and year 3 cohorts affected. MD Projects were impacted by university and research institute laboratory closures, limitations on travel and social distancing requirements. Necessary restrictions at clinical locations adversely impacted clinical projects, such that many could not continue. Direct face-to-face data collection could not occur, and there was substantial participant attrition or loss of data quality due to moving data collection online [5]. Added to this were significant delays in Ethics Committee review and approval [6] due to the large number of research projects needing revision. Furthermore, the project supervisor pool was significantly impacted, with many supervisors bearing increased clinical and academic workloads [7], whilst others were affected by the travel restrictions. In response to these challenges, it was necessary for the MD Project team to respond and oversee the rescope of many MD Projects in a short timeframe, so that the learning objectives of the MD Project Program were not compromised.

Given the breadth of adverse impacts and the need for repeated community quarantine periods, important questions arise about the success and quality of outcomes of the MD Projects undertaken during the COVID-19 pandemic.

Therefore, this study was undertaken to:

1. Determine the impact of COVID-19 on medical student research projects
2. Document the measures taken to rescope projects and support students in meeting the learning objectives of the program
3. Compare project outcomes and quality of project reports with previous years

### Outcomes

This report informs practice to protect student research project viability and support student learning and key academic outcomes, in response to a major external threat, including current and future pandemics.

## Methods

### Ethics Statement

This project was approved by the University of Sydney HREC #2016/457.

### Data Collection

#### Records of COVID-19 Impacts on MD Projects

Student cover letters and supervisor endorsement letters attached to MD Project final reports from cohorts completing MD Projects in 2020, 2021 and 2022 were reviewed. These documents identify any problems that arose during the student’s MD Project for consideration by the examiners. The letters were searched to identify the effect of COVID-19 on each project, with a checklist of key issues to be identified including COVID-19-related project delay, project downsizing, change in project within same topic or requirement for a new project. Findings were tallied and analysed by project type (Table 2). Where projects changed, the new project types were recorded and the rescoping plan documented. Accounts of students or staff being affected by COVID-19 illness and isolation were also recorded.

#### MD Project Grades by Year

Student grades for the MD Project final report for 2020–2022 were compared with grades for submission in 2017–2019, to assess the impact of COVID-19 on quality of reports and project outcomes.

### Data Analysis

Descriptive statistics and categorical analysis with the Chi-squared test were used to analyse the data. Grade data across the years was analysed using ANOVA, with a post hoc Tukey test. All data analyses were conducted in SPSS v26 (IBM, Armonk NY), and *p* < 0.05 is accepted as statistically significant.

## Results

### Cohort Student and Supervisor Numbers

**Fig. 1:**
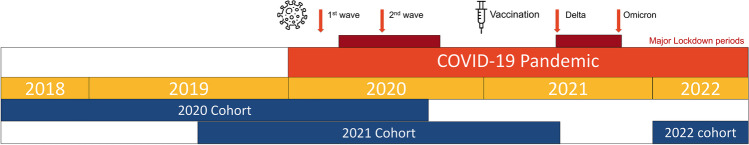
Timeline of COVID-19 effect on MD Projects, in Sydney, NSW, Australia, showing major infection waves, implementation of vaccination and government-imposed restriction periods [3]. Three student cohorts (2020–2022) were affected, and COVID-19 impacted projects at various stages of their progress

The COVID-19 pandemic impacted students at different phases of their research projects, from the early design phase, undertaking data collection, data analysis and final reporting stages. Both year 2 and year 3 student cohorts were affected from 2020 to 2022 (Fig. 1). The numbers of students and supervisors involved in the MD Project each year are shown in Table 3.

**Table 3:** Student and supervisor numbers

**Cohort year**	**Students (*n*)**	**Supervisors (*n*)**
2020	237	136
2021	267	126
2022	256	150
**Total**	**760**	**412**

### Number of Projects Affected by COVID-19

Over the 3-year period, 30 projects (6%) were changed due to reasons other than COVID-19, including failure of the original project at any stage (5 projects, 16%), delays in research ethics approvals (4 projects, 13%) and student-supervisor relationship issues (21 projects, 70%). This proportion did not differ significantly from previous years.

**Fig. 2:**
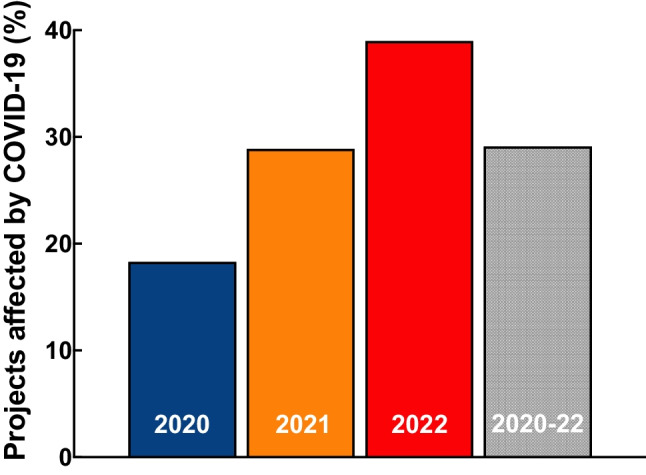
Proportion of all MD research projects affected by COVID-19 from 2020 to 2022 by year and averaged for the triennium

Over 2020–2022, 3-year period, between 20 and 40% of MD Projects were identified as being affected by COVID-19 (Fig. 2). This includes project delays, downsizing, projects rendered unviable and where students or their supervisors were affected by COVID-19 illness and isolation. The latter was especially significant for the 2022 cohort, who undertook projects during the February–April 2022 COVID-19 omicron variant wave, when 12% of the students were affected by COVID-19 illness and/or associated isolation requirements.

### Outcomes for COVID-19-Affected Projects

**Fig. 3:**
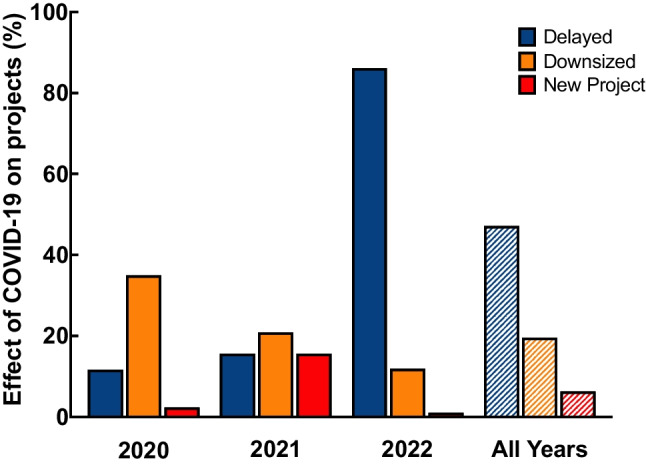
Outcome of COVID-19 on project progress from 2020 to 2022. Projects were affected by delays or downsizing, some projects were rendered unviable, and students needed to be moved to a new project

Among the 222 projects identified as adversely impacted by COVID-19, approximately a third progressed as expected. However, over the 3-year period, nearly half of COVID-19 impacted projects were delayed, 19% were downsized, and 6% were rendered not feasible and needed a completely new project (Fig. 3).

Overall, of the projects reported to be affected by COVID-19, two-thirds were impacted, with only a quarter still running to schedule. Outcomes by project type are shown in Table 4. Interestingly, about half of the prospective clinical studies went to plan, the rest were significantly delayed or downsized. Retrospective studies and laboratory studies (which are also prospective by nature) were significantly delayed. Projects involving analysis of existing datasets or the published literature were less affected overall, but where affected, were subject to significant delay.

**Table 4:** Outcomes for COVID-19-affected projects by project type

**Project type**	All projects *n* (%)	Projects affected by COVID *n* (%)	COVID-19 affected projects			
			No effect on project progress *n* (%)	Project delayed *n* (%)	Project downsized *n* (%)	Project changed to new project *n* (%)
**Prospective study**	138 (18)	80 (58)	43(54)	11 (14)	20 (25)	6 (8)
**Retrospective study**	288 (38)	70 (24)	6 (9)	48 (69)	11 (16)	5 (7)
**Laboratory project**	70 (9)	36 (51)	5(14)	20 (56)	10 (28)	1 (3)
**Dataset analysis**	55 (7)	10(18)	4 (44)	2 (22)	2 (22)	1 (11)
**Systematic review**	83 (11)	9 (11)	1(11)	8 (89)	-	-
**Product development**	24 (3)	8 (33)	1 (13)	6 (75)	-	1 (13)
**Study protocol**	15 (2)	4 (27)	0	4 (100)	-	-
**Narrative review**	69 (9)	2 (3)	0	2 (100)	–	-
**Scoping review**	18 (2)	3 (17)	0	3(100)	–	-
**Total**	760 (100)	222 (29)	60 (27)	104 (47)	43 (19)	14 (6)

Data is for all years (2020–2022 combined) showing the proportion of the different project types, those affected by COVID-19 and the outcomes of COVID-19 affected projects

**Fig. 4:**
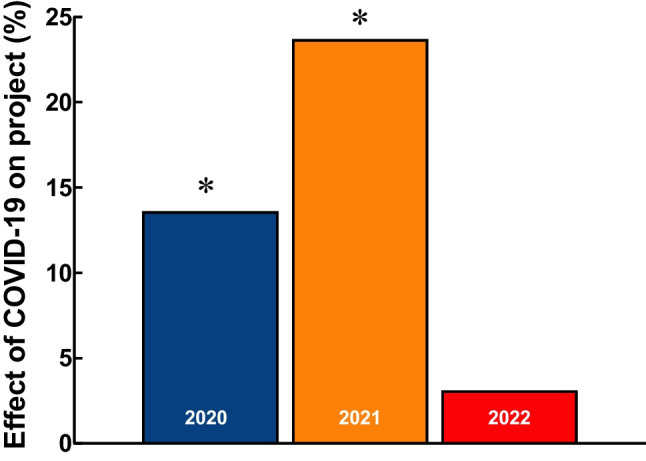
Percentage of projects requiring complete change by year. Significantly less projects needed to be changed due to COVID-19 in 2022. *p < 0.001, *χ*^2^-test, COVID-19-affected projects vs project change

There were a significantly greater number of projects that needed to be moved to new projects in the 2020 and 2021 cohorts, compared to 2022, due to a change in policy for the 2022 cohort, requiring all projects to either be feasible under the pandemic conditions or have a COVID-19 contingency plan (Fig. 4).

### Project Rescoping

Major rescoping plans implemented were negotiated between the student, supervisor and the MD Project team. Project rescoping options included considering the stage at which the student was at in the project and impact of COVID-19 on the project location and supervision capacity. Common rescoping strategies employed are shown in Table 5.

**Table 5:** Major rescoping plans implemented

**Original project**	**Rescoped to**
Prospective studies *Clinical or laboratory based*	Study protocol and ethics documents Downsize to a pilot study Examine pre-existing datasets Scoping or Narrative literature review
Retrospective studies *Clinical audits or dataset analyses*	Study protocol and ethics documents Downsize to a pilot study Examine pre-existing datasets Scoping or Narrative literature review
Product development projects	Downsize to product needs analysis Narrative literature review
Systematic reviews*	Narrative literature review

*No systematic review projects were changed due to COVID-19; changes were due to other reasons

### MD Project Final Report Grades

The MD Project summative assessment task is a 3000-word scientific report. MD Project final reports are graded by an extensive academic staff workforce, with 150 + academics being involved. Reports are graded to an assessment rubric, and graders are supplied with benchmarking advice. Over the last 6 years, the grades for MD Project final reports are very consistent, and there was no difference between the pre-COVID-19 and during COVID-19 final report grades (Table 6).

**Table 6:** MD Project final report grades pre- and during COVID-19

	**Pre-COVID-19**			**During COVID-19**		
**Year**	**2017**	**2018**	**2019**	**2020**	**2021**	**2022**
***n***	292	320	270	235	267	255
**Mean (SD)**	72.7 (10.1)	73.7 (10.8)	74.1 (9.4)	74.5 (9.5)	72.2 (10.1)	74.1 (10.6)
**Median (range)**	74 (38–95)	75 (10–93)	75 (42–91)	76 (45–93)	73 (25–93)	76 (0–91)

## Discussion

This is the first study to describe the effect of the COVID-19 pandemic on medical student research projects at an Australian University. The rapidly changing situation from March 2020 required research projects to be modified to retain feasibility and to meet educational goals. Common project problems were identified, and contingency plans were made, considering the stage of the project. Communication with all stakeholders was crucial and a multi-modal approach was needed, along with consistent messaging and reassurance. Despite significant disruption to the Australian health research workforce [1] and the Australian University sector [2], contingency plans were implemented to ensure students completed their MD Projects on time and to the required standards.

The COVID-19 pandemic had wide ranging impacts across the world. Australia, being an island nation had the advantage of geographic isolation. Government directed border closures and hotel-based quarantine for arrivals slowed the importation of the virus from international travellers. The Sydney Medical Program delivers MD Projects across both metropolitan and rural locations across New South Wales. This is Australia’s most populated state, with about 8 million residents, where in 2020–2021, the pandemic setting was of relatively low transmission, with a well-functioning contact tracing system in place [4]. During this period, there were three separate cohorts of students engaged in MD Projects; COVID-19 restrictions and later waves of infection occurred when students were at different phases of the research process, some were affected at the outset, others not until later in the research cycle. In addition, the 2020 and 2021 cohort had projects running on top of the core MD Program curriculum over 2.5 years; however, a Degree Program change in 2020 meant the 2022 cohort completed their projects in a 14-week block. Thus, there were distinct differences between the 2022 cohort and the earlier cohorts which were not only due to the pandemic.

### Common Project Problems

Whilst all project types were affected by delays and downsizing, those most affected were retrospective clinical audits (91%), due to reduced access the hospital medical records required and especially where the project supervisor was a frontline healthcare professional treating and caring for patients with COVID-19. As reported by others, there was a general lack of staff and student preparedness for the abrupt transition to remote learning [7, 8] with research work and project-based learning being particularly affected [5, 9]. Laboratory-based projects were halted during lockdown periods and were vulnerable to the transport delays caused by the pandemic. Laboratory reagents and consumables were in short supply, so the majority of the laboratory-based projects were delayed significantly, a problem that was experienced globally [10]. There is a growing literature describing pivoting from in person and laboratory based research projects to online modules and focus on research design and critical thinking [11–15]; however, the purpose of the medical student research project is to provide a hands-on experience, with in-depth exposure to research environment that is authentic, not a series of online modules. Interestingly, in this study, prospective studies were delayed and downsized, rather than being rendered non-viable, and whilst 80% were affected by the COVID-19 pandemic, and 50% went to plan anyway, suggesting there were inbuilt contingencies in these project types to protect recruitment and data collection.

### Practice Points

The MD Project team identified early (from March 2020) all projects affected or likely to be affected by COVID-19 and rapidly responded to develop rescope plans in negotiation with each student and project supervisor. Communications from the MD Project team were critical; we used cohort wide announcements through the online learning management system and direct email and short videos to communicate with students and supervisors. All project-related communications between students and supervisors were moved online. The importance of rapid and clear communication, featuring consistent, regular messaging to all stakeholders and using multi-modal approach (email, video and website) is well understood [7, 16]

The MD Project team designed a suite of project rescoping options that were feasible and fitted the requirements of the MD Project learning objectives and assessment. These options were presented to students via short videos and rescoping plans were reviewed by the MD Project team on a case-by-case basis. Effective communication and support for students and their safety and well-being were principal considerations in the project rescoping process. Medical students worldwide were reported to be anxious and stressed [17, 18], and the MD Project team and research supervisors realised the need to provide academic, social and moral support. It should be noted that the case-by-case nature of rescoping was a response to the situation as it unfolded, and the workload was substantial. Other reports also note case-by-case rescoping was unsustainable in the long term [7], but necessary at the time.

Rescoping plans were based on some key questions [9], primarily whether the original research question(s) could be answered, are hypotheses still testable and can data collection be continued safely? Rescoping decisions also considered the stage of the student project when COVID-19 effects were felt. COVID-19 restrictions and later waves of infection occurred when students were at different stages of the research process; some projects were affected at the outset, others not until the middle or near completion of the project.

Where possible, data collection and analysis for research projects was conducted remotely. Qualitative interview and focus group data were collected online by videoconferencing. Whilst this is necessary, it is noted that it does change the data collection design [5]. Participant recruitment was affected due to a lower ability and willingness of participants to contribute due to the pandemic [1]. Ethics committees struggled with the load of project amendments required for research projects to continue, and there were substantial delays in modification approvals [1, 6].

### Major Project Rescoping Strategies Employed

#### Writing up the Project as a Pilot Study

Where data collection was incomplete, students were allowed to write up the project as a pilot study, with the necessary considerations of the limitations due to insufficient statistical power. Many projects were significantly downscaled to pilot studies or studies based on data already collected by the research unit.

#### Converting to a Literature Review

Some projects could be rescoped to a systematic review and some to scoping reviews, using Preferred Reporting Items for Systematic Reviews and Meta-Analyses (PRISMA) methodology and guidelines [19, 20]. Other projects were moved to a structured narrative review of the topic area, which needed to include a systematic literature search strategy, data inclusion/exclusion criteria and a critical appraisal of the included literature.

#### Developing a Study Protocol

Many projects were still at the design stage, so a study protocol template was deployed, based on external grant and research ethics approval application forms. Students were allowed to complete the project by delivering a research study protocol that included scholarly consideration of the project rationale, a needs analysis and justification of the research methodology and expected outcomes.

#### Analysis of Publicly Available Health Data

Students with very severely impacted projects were offered a new project based on an existing, publicly available database including the Public Health Information Development Unit (PHIDU) database [21] and the National Health Survey data from the Australian Bureau of Statistics [22]. These projects required data cleaning and statistical analysis, usually involving regression models. Students required significant support with navigating the online databases and access to and training for appropriate statistical analysis software, so these projects required the assistance of a statistician for them to be viable.

Other positive outcomes included rescoping to projects related to COVID-19 and contributing to further understanding of the pandemic and a re-focus from collecting data to more critical thinking elements of the project process and research methodology [12]. Overall, the grades for MD project final reports were very consistent, and there was no difference between the pre-COVID-19 and during COVID-19 final report grades. This indicates there was no reduction in quality of project reports due to COVID. Although the effect of COVID on individual projects was considered by examiners, there was no amendment to the marking rubric or benchmarking which reveals that the quality of the student reports was maintained despite the pandemic.

### Limitations

This data is from a single medical school, from Australia, where the experience of the pandemic was mild in comparison to other countries with respect to COVID-19-related morbidity and mortality, though Australians were subject to very strict public health stay at home orders [23]. The data regarding COVID effects on the projects was self-reported, in report cover letters, and may not comprehensively articulate the effects of COVID on the project, particularly where projects were minimally delayed or downsized.

### Future Directions

Despite advances in vaccination and implementation of non-pharmaceutical interventions, such as social distancing and mask wearing, COVID-19 still presents significant challenges for both education and research, in both the clinical and laboratory environment. There are several areas in which research programs can build in resilience to the impacts of the pandemic or other external threat. Improving digital literacy overall and specifically regarding online publicly available health-related datasets, online survey tools and interviewing options would assist. Improving student access to statistical analysis software and the training to use such tools would be beneficial [24]. Considering options for projects that can be conducted without an ethics committee review or specific support for preparing amendments to research ethics applications would also be useful, not only during pandemic conditions. Support from Human Research Ethics Committees to expedite research project modifications is also needed, though this was a challenging time for Human Research Ethics Committees [25].

Medical students are considerably impacted by the COVID-19 pandemic, both personally and with respect to their education, and this situation will likely continue into the medium term, as further outbreaks of disease could occur [4]. When recruiting new projects to the program, there is a need for flexibility in the project offerings and outcomes and a contingency plan for projects that may not be feasible in the event of escalating problems due to the pandemic.

## Summary and Conclusions

We achieved a feasible rescope or reassignment of all affected projects such that the learning objectives for the MD Project were not compromised and overall grades were not impacted by the pandemic or related project rescoping. Further, we have learnt to build in pandemic resistant contingency plans for all future projects, which will also safeguard against other unexpected external threats.

## Data Availability

The datasets generated and/or analysed during the current study are not publicly available, as per conditions of Ethics Committee approval, but are available from the corresponding author on reasonable request.
